# Photoperiod-driven testicular DNA methylation in gonadotropin and sex steroid receptor promoters in Siberian hamsters

**DOI:** 10.1007/s00359-025-01733-w

**Published:** 2025-02-15

**Authors:** Irem Denizli, Ana Monteiro, Kathryn R. Elmer, Tyler J. Stevenson

**Affiliations:** Present Address: School of Biodiversity, One Health and Veterinary Medicine, College of Medical, Veterinary & Life Sciences, University of Glasgow, Glasgow, G12 8QQ UK

**Keywords:** Photoperiod, DNA methylation, Siberian hamsters, Mammals, Reproduction, Epigenetic regulation

## Abstract

Seasonal cycles in breeding, often orchestrated by annual changes in photoperiod, are common in nature. Here, we studied how change in photoperiod affects DNA methylation in the testes of a highly seasonal breeder: the Siberian hamster (*Phodopus sungorus)*. We hypothesized that DNA methylation in promoter regions associated with key reproductive genes such as follicle-stimulating hormone receptor in the testes is linked to breeding and non-breeding states. Using Oxford Nanopore sequencing, we identified more than 10 million (10,151,742) differentially methylated cytosine-guanine (CpG) sites in the genome between breeding long photoperiod and non-breeding short photoperiod conditions. ShinyGo enrichment analyses identified biological pathways consisting of reproductive system, hormone-mediated signalling and gonad development. We found that short photoperiod induced DNA methylation in the promoter regions for androgen receptor (*Ar)*, estrogen receptors (*Esr1*,* Esr2)*, kisspeptin1 receptor (*kiss1r)* and follicle-stimulating hormone receptor *(Fshr).* Long photoperiods were observed to have higher DNA methylation in promoters for basic helix-loop-helix ARNT-like 1 (*Bmal1)*, progesterone receptor (*Pgr)* and thyroid-stimulating hormone receptor (*Tshr)*. Our findings provide insights into the epigenetic mechanisms underlying seasonal adaptations in timing reproduction in Siberian hamsters and could be informative for understanding male fertility and reproductive disorders in mammals.

**Supplementary Information:**

The online version contains supplementary material available at 10.1007/s00359-025-01733-w.

## Introduction

Temperate zone animals exhibit remarkable physiological adaptations to seasonal changes in their environment, a fundamental attribute crucial for survival and successful reproduction. Environmental cues, such as the annual change in day length referred to as photoperiod, are proximate for timing of seasonal rhythms in animal reproduction, energy metabolism, and development (Gwinner 2003; Yoshimura 2010; Nakane and Yoshimura 2019; Beltrán-Frutos et al. 2022; Liddle et al. 2022; Stevenson et al. 2022). Other environmental factors, such as temperature, provide supplementary information that are integrated and fine tune timing of seasonal life-history transitions (Heideman and Bronson 1994; Wingfield 2007; Stevenson et al. 2022).

Photoperiodic cues have a critical role in timing phases of reproduction and testicular development in seasonal breeders (Gaston and Menaker 1967; Konishi 1967; Elliott et al. 1972). In many summer breeding mammals, long photoperiod promotes gametogenesis and sex steroid synthesis in gonads whereas short photoperiod causes reproductive involution (Hegstrom and Breedlove 1999; Körtner and Geiser 2000; Young and Nelson 2000; Pyter et al. 2005; Moffatt-Blue et al. 2006). For seasonal breeders the hypothalamic-pituitary-gonadal (HPG) axis governs seasonal changes in reproduction (Bédécarrats 2015; Kaprara and Huhtaniemi 2017). The kisspeptin receptor (*Kiss1r*) is a key regulator of reproductive function through its role in stimulating gonadotropin-releasing hormone (GnRH) secretion (Harter et al. 2018). Follicle-stimulating hormone (FSH) and luteinizing hormone (LH) are released from the pituitary gland in response to stimulation by GnRH (Kazmi and Can 2024). When stimulated by LH, testosterone is produced by Leydig cells in the testes, playing a vital role in spermatogenesis maturation and gametogenesis (Zirkin and Papadopoulos 2018; Oduwole et al. 2021). FSH acts on Sertoli cells within the seminiferous tubules, supporting germ cell development and spermatogenesis (Griswold 1998; Walker and Cheng 2005; Oduwole et al. 2021).

The Siberian hamster (*Phodopus sungorus)*, also known as the Djungarian hamster, is known to exhibit a wide range of physiological and behavioural changes in response to changes in photoperiod (Lewis and Ebling 2018). Siberian hamsters housed in long day (LD) conditions, imitating the summer day lengths, have increased gonad size, spermatogenesis, follicular maturation and steroidogenesis thus enhancing reproductive success (Duncan and Goldman 1984; Salverson et al. 2007; Lynch et al. 2016). Gonadal regression occurs in response to short day (SD) conditions, akin to winter day lengths (Heldmaier et al. 1981; Greives et al. 2007; Lewis and Ebling 2018). This results in decreased gonadal size and reduced reproductive behaviour, such as copulatory behaviour or nesting. The regulation of gonadal size and function in Siberian hamsters in response to photoperiod remains a complex and incompletely understood phenomenon. While the hypothalamus is recognized as a central player in orchestrating seasonal transitions in reproductive physiology, the underlying genes and signalling pathways involved in seasonal plasticity in the testes are poorly described. Identifying the specific genes modulated by photoperiodic cues within the gonads represents an important area of scientific investigation, holding promise for a deeper understanding of the molecular basis of environmental regulation of reproductive physiology in these seasonal breeders.

Changes in photoperiod trigger epigenetic modifications that influence reproductive processes in plants (Cortijo et al. 2014; Ai et al. 2021; Mahmood et al. 2023), insects (Meuti and Denlinger 2013; Pegoraro et al. 2015), birds(Tolla and Stevenson 2020; Lindner et al. 2021; Liu et al. 2023) and mammals (Alvarado et al. 2015; He et al. 2023). One important epigenetic process underpinning the regulation of reproduction in response to changing day lengths is photoperiod-driven changes in DNA methylation (Stevenson 2017a, b). Responses to short and long photoperiods have been shown to cause differential expression patterns in genes targeting epigenetic regulators, including DNA methyltransferases (DNMTs) (Giannetto et al. 2013; Lynch et al. 2016; Coyle et al. 2020; Tolla and Stevenson 2020), and chromatin remodeling enzymes (Lynch et al. 2017; Borah et al. 2022). Rader et al. (2022) demonstrated that photoperiod exposure in Siberian hamsters alters the expression of m6A-related methyltransferase complex genes, such as *Mettl14*, *Wtap*, and demethylation-associated genes (*Fto* and *Alkbh5*), suggesting a role for RNA methylation in testicular responses to photoperiod and recovery during recrudescence. DNA methylation increased in short days in the wasp (*Nasonia vitripennis*) and was found to be necessary for the shift into diapause (Pegoraro et al. 2015). Siberian hamsters were reported to have increased expression of the genes *Dnmt3a* and *Dnmt3b* in uterine and testicular tissue and elevated DNA methylation genome-wide when exposed to short-day photoperiods (Lynch et al. 2016). Targeted analyses in the mediobasal hypothalamus have indicated that the *dio3* promoter is one region that is epigenetically regulated with higher DNA methylation in the long photoperiod versus short photoperiod state (Stevenson and Prendergast 2013). In humans, whole-genome sequencing in sperm has uncovered regions characterized by the enrichment of H3K4me3 and concurrent DNA methylation, indicating a functional interplay relevant to fertility and development (Lambrot et al. 2021; Dura et al. 2022a). Analyses of spermatogonia stem cells identified a crucial role of DNMT3A-dependent DNA methylation for spermatogenesis (Dura et al. 2022b). Aberrant sperm DNA methylation patterns have been identified as predictive indicators of male fertility status and embryo quality, with consistent differences observed between infertile and fertile men (Aston et al. 2015).

To better understand how photoperiod drives changes in the reproductive function of Siberian hamsters, this study investigated genome-wide DNA methylation changes in hamster testes. By using Oxford Nanopore MinION sequencing and Nanopolish bioinformatic tools, we examined the DNA methylation profiles of testicular methylomes from non-breeding short photoperiod and breeding long photoperiod conditions. We hypothesised that under short photoperiod conditions DNA methylation would be elevated in regions associated with reproductive genes and sex-steroid hormone synthesis related pathways. Our study identified highly methylated promoter regions in non-breeding states, including androgen receptor (*Ar)*, estrogen receptors (*Esr1*,* Esr2)*, kisspeptin-1 receptor (*kiss1r)* and follicle-stimulating hormone receptor *(Fshr).* Reciprocally high methylation in promoters for basic helix-loop-helix ARNT-like 1 (*Bmal1)*, progesterone receptor (*Pgr)* and thyroid-stimulating hormone receptor (*Tshr)* upon LD were also found.

## Materials and methods

### Animal Housing and Photoperiodic Treatment

Adult male Siberian hamsters, aged 3–8 months, were selected from a colony kept at the University of Aberdeen. The hamsters were kept under a long day (LD) photoperiod (15 L:9D) in cages made of polypropylene. Food and water were provided ad libitum along with cotton nesting material. The University of Aberdeen’s Animal Welfare and Ethics Review Board approved all procedures, and the study was conducted under an approved Home Office licence (70/7917).

In this study, twelve mature male hamsters were used. A group of six males hamsters were pseudorandomly selected from the colony and moved to cabinets in a short-day photoperiod (Arrowmight; SD 9D:15 L) for 8 weeks and served as the treatment group. Another group of six male hamsters were kept in the long day colony room and served as the photoperiodic control group. At the end of the study, the animals were killed by cervical dislocation, and the mass of their testicles was measured to ± 0.1 g using aeADAM scales (Adam Equipment PGL2002). Tissues were frozen in powdered dry ice and stored at -80 °C.

### DNA extraction

Genomic DNA was extracted from > 30 mg of testes tissue using Qiagen QIAmp DNA Mini Kit (Qiagen; catalog #51304 and #51306) following manufacturer’s protocol. Genomic DNA was purified with NaAcetate (3 M, pH 5.2) and precipitated with EtOH. Nucleic acid quantity and 280/260 values were determined using NanoDrop.

### Whole genome sequencing

Extracted genomic DNA was sequenced using Oxford Nanopore Minion and SQK-LSK109 ligation sequencing kit. Individual samples were identified using EXP-NBD104 native barcoding. First, 1.5–3 µg of gDNA was prepared in nuclease-free water. Equimolar amounts of each barcode were pooled in to produce 700 ng pooled library. The Flowcell (FLO-MIN106D) was primed and loaded onto the MinION platform, containing 12 samples comprising DNA from 6 LD- and 6 SD-treated hamster testes. Sequencing was done following the manufacturer’s protocol. The long-read sequencing lasted for 72 h at a voltage of − 180mV, and fast5 files were generated to facilitate downstream analyses.

### Bioinformatic pipeline and methylation calling

For bioinformatic analyses we first extracted the reads from FAST5 files by basecalling using *guppy*. The barcode trimming was done by using porechop (Wick et al. 2017). We mapped reads to the *Phodopus sungorus* reference genome (SUB13765567) with minimap2 (Loman et al. 2015a; Li 2018). Next, each file was indexed and used for methylation calling with NanoPolish(Loman et al. 2015b) with the LD group as “Control” and SD group as “Case” data. DNA fragments that were not aligned to chromosomes were excluded from further analyses. Methylated regions were filtered based on the *log_lik_ratio* values. Likelihood values more than 0 indicate methylation whereas lower values show unmethylation. We used log_lik_ratio > 2 to have strong evidence for methylation based on the suggestion of the developers on GitHub.

Chromosomal information was taken from the study of Moore and colleagues (Moore et al. 2022). We used *tidyr* and *dplyr* packages of R and visualized the genome-wide distribution of differentially methylated regions. With the *Phodopus sungorus* annotation, we were able to annotate the gene structures (gene body, exon, intron, promoter) using *AnnotationDbi* (Hervé Pagès Seth Falcon Nianhua Li 2017) and *valr* (Riemondy et al. 2017) package of R and analysed the methylation pattern changes in each region. To visualize the findings, *ggplot2* package for R, and (https://bioinformatics.psb.ugent.be/webtools/Venn/) were used for Venn diagrams. Finally, we used ShinyGO 0.76 (Ge et al. 2020) to perform Gene Ontology (GO) enrichment analysis on identified regions. The codes used for this study are stored on github (https://github.com/IremDenizli/TestesMethylation.git).

Principal component analysis (PCA) was performed to investigate the variation in testes mass across different photoperiods. The PCA was conducted using the *FactomineR* library in R (R version 4.4.1) with the *stats* and *ggplot2* packages for visualization.

### Statistical analysis

The statistical analyses were performed using R statistical software. T-tests were used to compare read counts. The package includes the statistical comparison function, *stat_compare_means()*, which use two-tailed t-tests by default.

## Results

### Photoperiodic variation in reproductive physiology

**Fig. 1:**
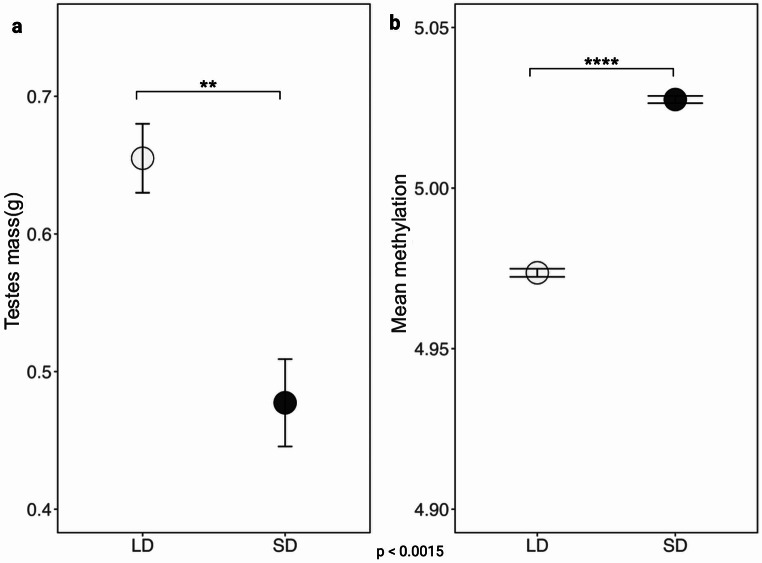
Comparison of testes mass and methylated sites between LD and SD groups in Siberian hamsters. T-test were used to calculate statistics. (**a**) Means plot represents the change in testes mass upon LD and SD treatments. Testes mass of SD treated hamsters was significantly lower than LD treated. (**b**) The graph illustrates the total count of methylated sites in LD and SD groups. SD treated hamsters have higher amount of overall methylation in testes tissues in contrast to LD hamsters

As expected, testicular mass significantly decreased in hamsters transferred to SD compared to LD conditions, with a mean reduction of 27% (*p* < 0.01, Fig. 1a).

### Short photoperiodic induced increase in testicular DNA methylation

Exposure to a short photoperiod resulted in a higher mean of likelihood supporting stronger DNA methylation (5.03 *±* 0.0011, 12502216 methylated CpGs) compared to a long photoperiod (4.97 *±* 0.0013, 10493712 methylated CpGs). The PCA revealed distinct clustering of samples based on photoperiods. The first two principal components (Testes Mass – Methylation) explained 68.5% and 31.5% of the variance, respectively. Samples treated with short photoperiod tended to cluster separately from the samples from those treated with long photoperiod (Suppl. Figure 1), suggesting that gonadal involution is associated with increased DNA methylation (Fig. 1b).

**Fig. 2:**
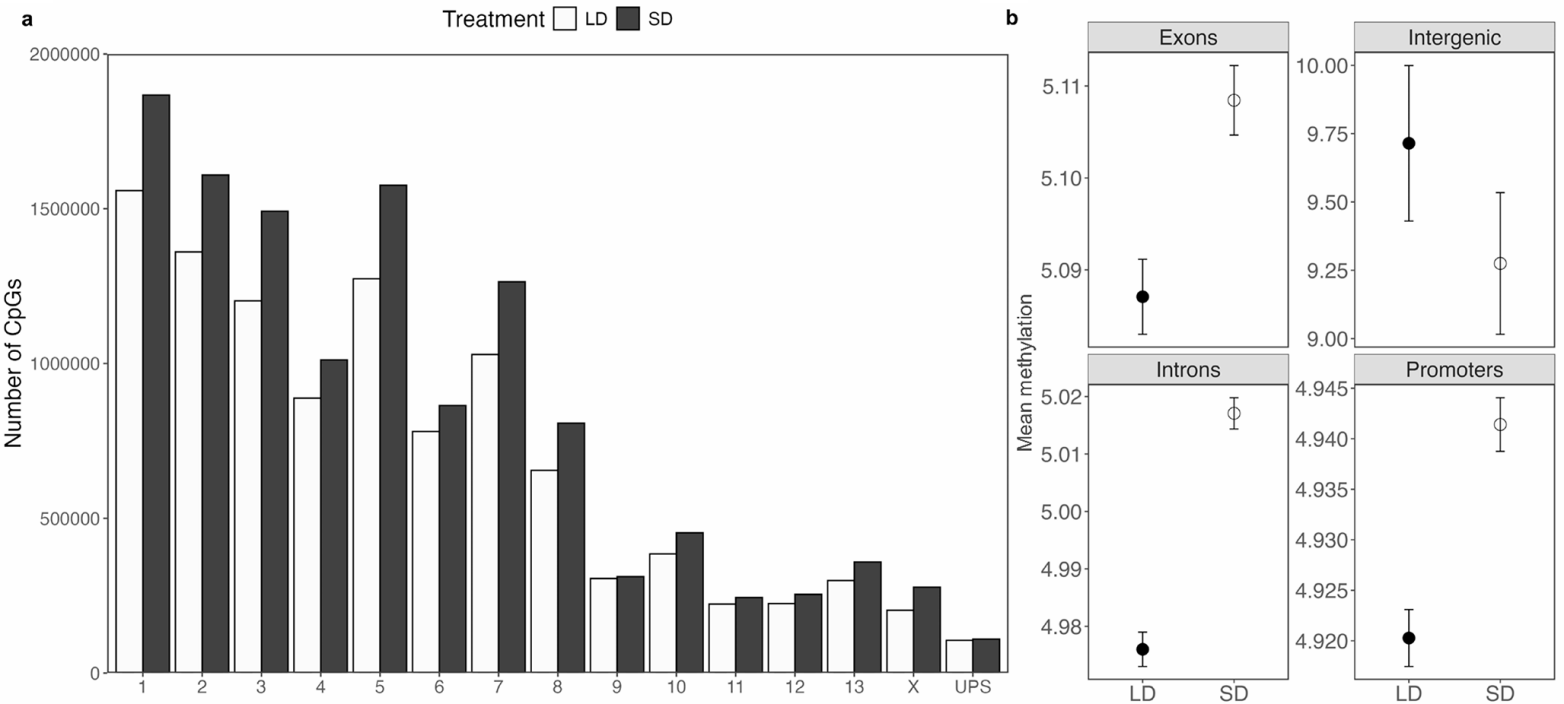
Analysis of genome-wide DNA methylation patterns in short day (SD) and long day (LD) hamster testes. (**a**) Genome-wide distribution of methylated CpG sites in SD and LD-treated hamsters. The graph illustrates the methylated regions across the entire genome for each treatment group, highlighting the differences in methylation patterns between the two treatment groups. (**b**) Methylated regions across promoters and gene bodies were analysed and were significantly higher in promoters. Promoters and gene bodies had higher methylation in SD compared to LD groups

Next, we found the amount of methylated CpG sites across the genome and mean LLR indicating methylation in different genetic regions (Fig. 2a and b). Chromosomes 1 through 8 exhibited significantly higher amount of methylated CpGs in the short photoperiod condition (Chromosome 1: 1867661 sites) compared to the long photoperiod condition (Chromosome 1: 1558531 sites). In contrast, Chromosomes 9 through 13, the X chromosome and the unplaced scaffolds (UPS) showed a similar distribution of methylated sites between the two treatments.

Then we annotated these regions and identified the amount of methylated gene bodies and promoters in both treatment groups individually (Suppl. Figure 2, Table S1 and Table S2). We found that exons had the highest levels of mean DNA methylation under short photoperiod, whereas intergenic regions have higher mean methylation under long photoperiod (Fig. 2b). We also showed that promoters show higher mean methylation under short photoperiod (2,861,379 sites) (Suppl. Table 1) compared to long photoperiod (2,594,755 sites) (Suppl. Table 1).

**Fig. 3:**
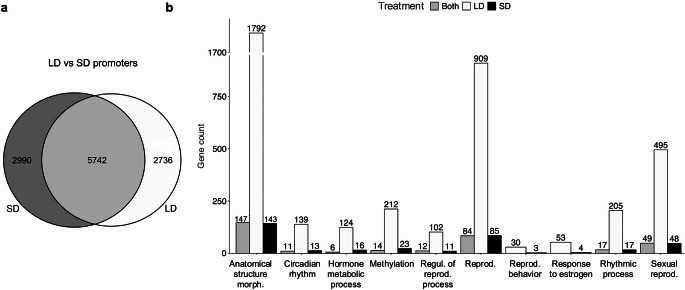
Gene ontology enrichment of differentially methylated promoters in testicular tissues of hamsters under different photoperiods. (**a**) Venn diagram illustrating the overlap and unique sets of differentially methylated promoters between LD and SD conditions. A total of 2736 promoters were unique to LD conditions (white), 2990 promoters were unique to SD conditions (dark gray), and 5742 promoters were common to both conditions (light gray). (**b**) Bar graph displaying the number of differentially methylated promoters involved in various biological processes under LD (white), SD (black), and both conditions (grey)

The analysis of differentially methylated promoters in testicular tissues of hamsters revealed significant photoperiod-driven methylation patterns. In total, 11,468 differentially methylated promoters were identified, with 2736 unique to long-photoperiod, 2990 unique to short-photoperiod, and 5742 common to both photoperiods (Fig. 3a). Gene Ontology analysis of these differentially methylated promoters highlighted distinct biological processes affected under LD and SD conditions (Fig. 3b). Promoters involved in anatomical structure morphogenesis, multicellular organism reproduction, and sexual reproduction showed significant methylation changes, with a higher number of differentially methylated promoters under LD. Processes such as circadian rhythm, hormone metabolic process, methylation, and rhythmic processes had fewer differentially methylated promoters and exhibited more similar numbers between LD and SD.

### Targeted analyses of DNA methylation in selected genes involved in reproduction

**Fig. 4:**
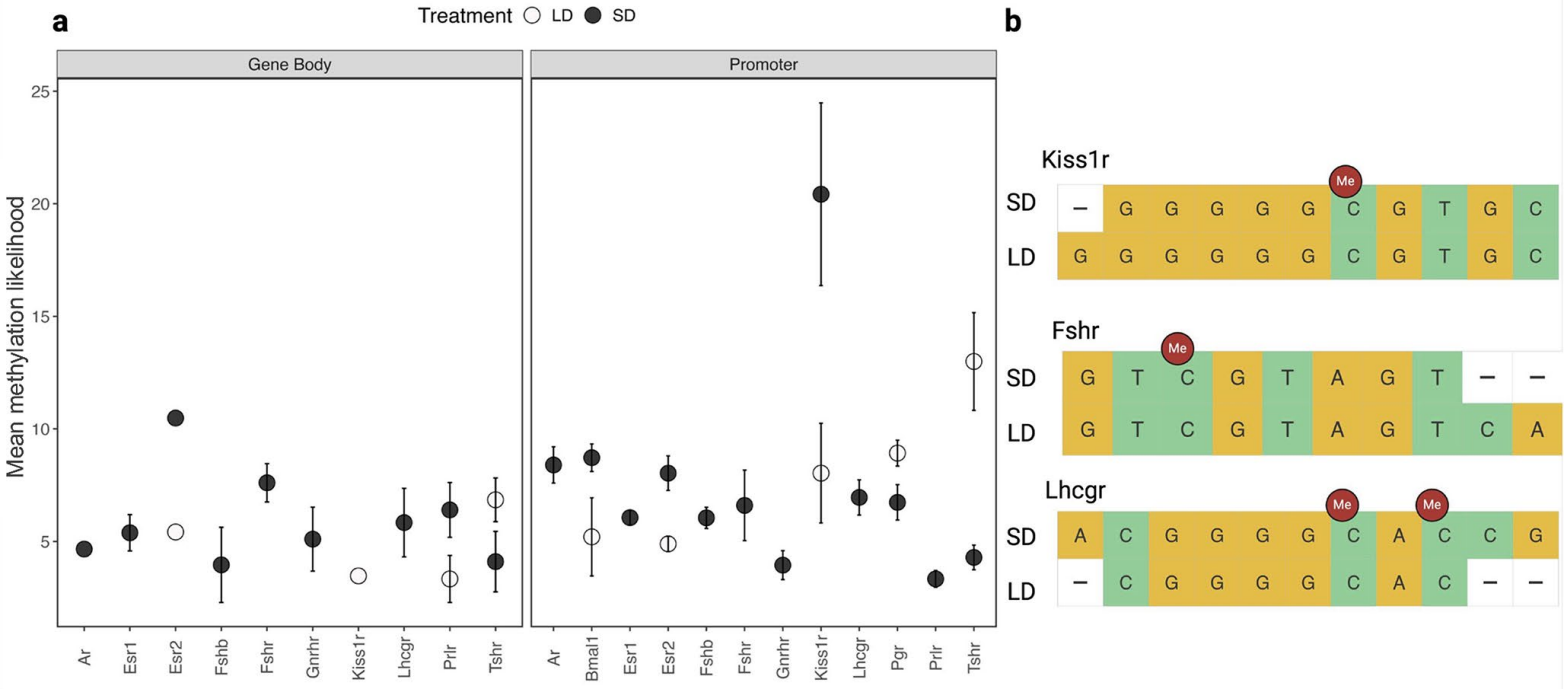
Methylation frequency of reproduction-related gene bodies and promoters. (**a**) The figure displays the methylation frequency observed within the gene bodies and promoters of reproduction-related genes in the testes of Siberian hamsters. (**b**) Schematic showing methylated cytosines in *Kiss1r*, *Fshr* and *Lhcgr*

In our targeted epigenetic analysis of genes implicated in the regulation of reproductive functions, we examined the methylation patterns in both gene bodies and promoters of several key genes, including estrogen receptors (*Esr1* and *Esr2*), androgen receptor (*Ar*), gonadotropin-releasing hormone receptor (*Gnrhr*), and thyroid-stimulating hormone receptor (*Tshr*), among others (Fig. 4). Our findings indicate that exposure to SD drastically induced methylation changes in the promoters of *Esr2* and *Kiss1r*. Specifically, the mean methylation frequency in the *Esr2* promoter was 7.1 across 69 sites in SD, and for the *Kiss1r* promoter, it was markedly elevated at 14.6 across 7 sites. Conversely, LD exposure resulted in increased methylation in the promoters of *Bmal1*, *Tshr*, and *Pgr*, with mean methylation frequencies of 5.68 (6 sites), 9.6 (7 sites), and 6.3 (58 sites), respectively. Further examination revealed differential methylation levels in other gene promoters. For instance, under short photoperiod the *Esr1* promoter exhibited a mean methylation frequency of 5.81 across 145 sites, while the *Ar* promoter showed a mean frequency of 5.50 across 26 sites. In similar conditions, our data also highlighted notable methylation frequencies in the promoters of other genes such as *Fshb* (5.55 across 27 sites), *Fshr* (6.60 across 3 sites), *Gnrhr* (3.73 across 10 sites), and *Lhcgr* (5.06 across 19 sites).

## Discussion

Our investigation into DNA methylation patterns in Siberian hamster testes identified photoinduced changes at global and gene-specific levels. This study shows that short and long photoperiods induce distinct patterns of DNA methylation genome wide and on specific gene promoters associated with the control of seasonal reproduction. We discovered that only short photoperiod triggers methylation in *Ar*,* Esr1*,* Fshb*,* Fshr*,* Gnrhr*,* Lhcgr and Prlr* promoters whereas long photoperiod induced methylation in *Tshr and Pgr* promoters. Notably, *Kiss1r* promoter showed an enhanced methylation under short photoperiod. These differential methylation patterns reveal that epigenetic modifications are critical for the genomic regulation of seasonal reproduction.

Photoinduced changes in DNA methylation is emerging as a common genomic mechanism involved in reproduction. In insects, such as *Nasonia vitripennis*, an elevation in DNA methylation levels was observed during shorter day conditions and was demonstrated to be crucial for the induction of diapause (Pegoraro et al. 2015). Similarly, in plants, photoperiodic cues regulate DNA methylation patterns that control flowering time and reproductive success. In species such as *Arabidopsis thaliana*, methylation of flowering locus T was associated with late flowering even under inductive long photoperiod, indicating the role of epigenetic regulation in reproduction (Zicola et al. 2019). Additionally, DNA methylation may play a crucial role in the regulation of reproduction and migration in birds. In redheaded buntings *dnmt3a* was found to be higher in the migratory state compared to the non-migratory (Sharma et al. 2018). In another study in great tit (*Parus major)*, it was reported that changes in DNA methylation patterns within specific genes coincide with the initiation of egg laying (Lindner et al. 2021). For example, hypomethylation of CpG sites within the promoter regions of genes like *MYLK-like* and *GP2-like* occurs in the weeks prior to laying, suggesting that DNA methylation changes are associated with the onset of reproductive events. Furthermore, European starlings were found to increase DNA methyltransferase activity in the preoptic area of the hypothalamus during non-breeding compared to breeding conditions (Stevenson et al. 2012). Previous work in Siberian hamsters has shown that differential methylation patterns in testes may cause the photoperiod-driven alteration in reproduction.Lynch et al. (2016) identified increased global DNA methylation in the testes and higher DNA methyltransferase (*dmt3a)* expression in SD-treated group compared to LD-treated group indicating the photoperiod-driven changes in methylation patterns.

In this study we observed increased testicular DNA methylation in short day conditions and identified precise genomic motifs that exhibit differential methylation. Under short photoperiod, the promoters of *Ar*, *Bmal1*, *Esr1*, *Esr2*, *Fshb*,* Fshr*, *Gnrhr*, and *Prlr* exhibit methylation, suggesting a potential mechanism for epigenetic regulation of testicular function in Siberian hamsters.

Firstly, methylation of *Ar*, *Esr1*, and *Esr2* promoters, which encode androgen and estrogen receptors, respectively, likely reduces spermatogenesis (McLachlan et al. 2002; Oduwole and Huhtaniemi 2014; Huhtaniemi 2018) and gonadal development (Kobayashi et al. 2014; Fadlalla et al. 2017). Secondly, methylation of *Fshb*,* Fshr*, *Gnrhr*, and *Prlr* promoters, which are involved in the regulation of follicle-stimulating hormone, gonadotropin-releasing hormone, and prolactin receptors, respectively, may prevent spermatogenesis (McLachlan et al. 2002; Oduwole and Huhtaniemi 2014; Huhtaniemi 2018) and the synthesis of hormone signaling pathways required for reproduction (Lehman et al. 1997; Anand et al. 2002; Huhtaniemi 2015; Stewart et al. 2022). Methylation of these promoters under short photoperiod conditions may cause disruption in reproductive pathways in male hamsters, leading to aberrant levels of reproductive hormones, disturbances in reproductive cycles, and diminished fertility.

Kisspeptin receptor (*Kiss1r*) and its ligand kisspeptin (encoded by *Kiss1*) are involved in the regulation of both reproductive function and energy balance. Kisspeptin signaling via its receptor is essential for stimulating gonadotropin-releasing hormone (*GnRH*) secretion, thereby orchestrating reproductive hormone levels and reproductive processes (Xie et al. 2022). Specifically, kisspeptin neurons in the hypothalamus are implicated in the modulation of food intake and energy expenditure (De Bond and Smith 2014). Dysregulation of kisspeptin signaling can be linked to disruptions in both reproductive function and energy balance, highlighting the connection of these physiological processes. The increase in methylation of the *Kiss1r* promoter under short photoperiod compared to long photoperiod in testis tissue of hamsters suggests a potential epigenetic regulatory mechanism for the kisspeptin signalling pathway in response to changes in day length. The increased methylation of the *Kiss1r* promoter under short photoperiods may lead to reduced expression of the kisspeptin receptor in testicular tissue. Although the function of kisspeptin in the testes is unclear, the decreased methylation of the *Kiss1r* promoter under long photoperiods suggests a potential role in regulating spermatogenesis or steroidogenesis. These results are similar to observations in the ovary, where kisspeptin expression transiently increases after 8 weeks of long photoperiod exposure (Shahed and Young 2009). We observed that *Kiss1r* promoter methylation showed a higher variance than other promoters, suggesting more dynamic, fine-tuned epigenetic regulation at this locus. Lower DNA methylation in the kisspeptin receptor could reflect an adaptive mechanism that allows the gonads to rapidly respond to changes in environmental cues (e.g., nutrient variability). For example, positive energy balance could act via kisspeptin receptor signaling to enhance reproductive function via increased spermatogenesis and/or steroidogenesis (Schneider 2004; De Bond and Smith 2014).

Androgens are essential for reproduction (Vornberger et al. 1994). Research in mice has demonstrated that male mice lacking androgen receptors (*Ar*) experience impaired germ cell development and decreased testosterone levels, resulting in azoospermia and infertility (Xu et al. 2007). Sertoli cells play a crucial role in supporting testis development and spermatogenesis by providing necessary nutrients (Xie et al. 2023). Their maturation and proliferation are regulated through the androgen receptor signaling pathway (Wang et al. 2022). In Syrian hamsters, which also exhibit testicular regression under short photoperiods, exposure to reduced daylight has been linked to a decrease in the number of Sertoli cells (Martínez-Hernández et al. 2020). In our experiments, short photoperiod exposure in Siberian hamsters led to an increase in methylation in promoter of androgen receptors in testicular tissue.

We then observed a significant increase in methylated promoters for genes such as *Pgr* and *Tshr* in testes tissue from hamsters housed in long photoperiods. Progesterone receptor knockout (PRKO) mice were shown to have testes that exhibit noticeable enlargement compared to those of wild-type control mice (Lue et al. 2013). Additionally, PRKO mice produce more sperm and have higher numbers of Sertoli and Leydig cells. Moreover, males lacking the progesterone receptor show decreased levels of follicle-stimulating hormone (FSH) and increased levels of inhibin, without significant alterations in testosterone levels or testicular morphology (Schneider et al. 2005). Previous work supports the conjecture that progesterone and its signalling components can modulate androgenic pathways. For instance, progesterone receptor antagonists have been shown to enhance *Ar* expression in vitro (Narvekar et al. 2004) and androgen receptor activity has been reported to regulate progesterone receptor expression and protein levels in endometrial tissues (Babayev et al. 2017). While classical progesterone receptor signalling appears dispensable for male fertility, evidence points to membrane progesterone receptors as potentially essential modulators of testicular function (El-Hefnawy and Huhtaniemi 1999). These complex interactions may be particularly relevant in photoperiodic rodents, as earlier studies in golden hamsters have demonstrated shifts in testicular steroid profiles and function linked to seasonal changes. In their study, Bartke et al. (1990) revealed that short photoperiod exposure in hamsters causes testicular regression, reduces testosterone production, and promotes a compensatory increase in progesterone secretion. Taken together, these observations suggest that progesterone could influence *Ar*-mediated processes during seasonal reproductive transitions.

We also identified the pathways that might be affected by photoperiod-driven methylation in testicular tissue by Gene Ontology (GO) analysis. Long photoperiod mostly induced methylation in promoters of genes that play role in anatomical structure morphogenesis and reproduction, respectively. Genes that play role in circadian rhythm and rhythmic process pathways have distinct numbers of promoters that are methylated under long photoperiod. LD induced methylation in promoters of *clock* and *per1* whereas SD induced methylation in promoter of *cry1* (Supp. Table 3). This indicates that photoperiod-driven methylation likely plays a crucial role in regulating the timing and synchronization of reproductive activities in hamsters by modulating key genes involved in circadian rhythms and reproductive processes in testicular tissue.

One major limitation of this study was an inability to assemble the Y chromosome, which may have important implications for understanding sex-specific effects on DNA methylation. The human Y chromosome, being one of the smallest and least gene-rich chromosomes, has posed significant challenges for sequencing and assembly. Until recently, the Y chromosome remained one of the last human chromosomes to be fully sequenced due to its highly repetitive and palindromic nature (Skaletsky et al. 2003; Miga et al. 2014; Vollger et al. 2022). The repetitive sequences make it difficult to assemble using traditional sequencing methods. Only with recent advances in sequencing technologies and bioinformatic tools have researchers been able to overcome these challenges and assemble the Y chromosome accurately. Therefore, it is not surprising that our study encountered difficulties in assembling the Y chromosome. The next steps should focus on developing bioinformatic pipelines that will enable the ability to resolve this issue and consequently provide a detailed understanding of the epigenetic mechanisms underlying seasonal reproductive adaptations. Additionally, while our study provides valuable insights into DNA methylation patterns under short photoperiod in Siberian hamster testicular tissue, the lack of gene expression data limits our ability to directly correlate methylation changes in gene expression. Further investigation into the transcriptional activity of identified genes will be crucial for elucidating the functional consequences of photoperiod-driven methylation changes on reproductive physiology in Siberian hamsters.

In conclusion, our investigation into DNA methylation patterns in Siberian hamster testes under different photoperiods provides new insights into the epigenetic regulation of seasonal reproduction. We found that short photoperiods induced methylation on key gene promoters including *Kiss1r*,* Ar*,* Esr1-2*, and *Bmal1*, implicating the importance of epigenetic modifications in these genes for the control of seasonal fertility.

Conversely, long photoperiods induce methylation on promoters of genes such as *Pgr and Tshr*, indicating tissue specific downregulation of progesterone and thyrotropin-stimulating hormone signaling pathways. These findings underscore the dynamic interplay between epigenetic modifications and gene expression in response to environmental cues, contributing to our understanding of the evolutionary strategies employed by mammals to optimize reproductive success.Tolla and Stevenson (2020) previously reported that male and female animals show different physiological responses to same photoperiod condition. Next steps should be to consider whether similar changes occur in ovarian and uterine tissue. Increased understanding of epigenomic and transcriptomic changes in testicular, ovarian, and uterine tissue will be instrumental in unraveling the complex mechanisms underlying seasonal reproductive adaptations and fertility.

## Electronic supplementary material

Below is the link to the electronic supplementary material.

Supplementary Material 1: Table 1 Promoters that are methylated upon LD and SD treatment.

Supplementary Material 2: Table 2 Gene Ontology results of the methylated promoters under long photoperiod and short photoperiod.

Supplementary Material 3: Table 3 Mean log-likelihood ratio of the chromosomes after long- and short-photoperiod treatments. Higher LLR supports stronger methylation.

Supplementary Material 4: Table 4 Mean LLR of each gene upon SD and LD condition.

Supplementary Material 5: Table 5 Significance results of gene of interests.

Supplementary Material 6: Figure 1 PCA result of samples.

Supplementary Material 7: Figure 2 Methylation frequency across chromosomes for each sample treated with different photoperiod.

## Data Availability

Sequences are pending accession code number.
