# Supplementary figures and images for: Photoperiod-driven testicular DNA methylation in gonadotropin and sex steroid receptor promoters in Siberian hamsters
Source: J Comp Physiol A Neuroethol Sens Neural Behav Physiol. 2025 Feb 15;211(3):327–37. doi: 10.1007/s00359-025-01733-w (PMC12081511; doi:10.1007/s00359-025-01733-w)

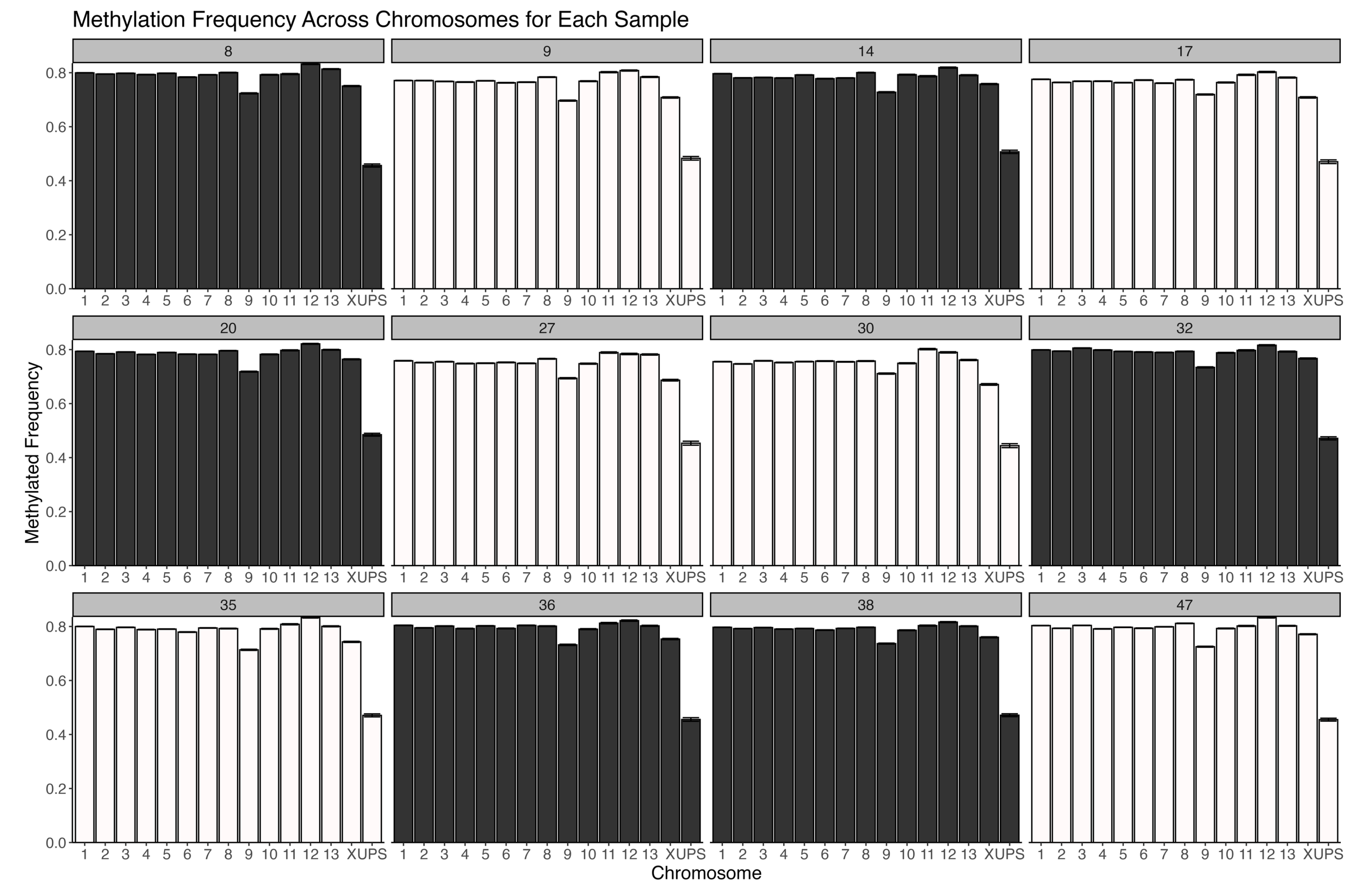

Supplement: Supplementary file 6 — Supplementary Material 6: Figure 1 PCA result of samples. [file 359_2025_1733_MOESM6_ESM.tiff]
